# Geographical and behavioral risks associated with *Schistosoma haematobium* infection in an area of complex transmission

**DOI:** 10.1186/s13071-018-3064-5

**Published:** 2018-08-25

**Authors:** Teckla Angelo, Joram Buza, Safari Methusela Kinung’hi, Henry Curtis Kariuki, Joseph Rogathe Mwanga, David Zadock Munisi, Shona Wilson

**Affiliations:** 1 Department of Global Health, School of Life Sciences and Bioengineering, Nelson Mandela African Institution of Science and Technology, P. O. Box 447, Arusha, Tanzania; 2 National Institute for Medical Research, Mwanza Centre, P. O. Box 1462, Mwanza, Tanzania; 3 Kenya Methodist University, School of Medicine and Health Sciences, P.O. Box 267, Meru, Kenya; 4 Department of Pathology, University of Cambridge, Tennis Court Road, Cambridge, CB2 1QP UK

**Keywords:** Urogenital schistosomiasis, Malacological surveys, GPS, Water contact behavior

## Abstract

**Background:**

*Schistosoma haematobium* infection in endemic areas varies depending on the nature and complexity of the transmission networks present. Studies of micro-geographical transmission of *S. haematobium* infection indicate that discrepancy in prevalence between households is associated with diverse water contact behaviors and transmission that is restricted to particular sites harboring snail intermediate hosts. Detection of variations in the transmission sources with complex transmission networks of water bodies is required for optimization of malacological control. Longitudinal parasitological and malacological surveys were conducted to investigate geographical variations in transmission of urogenital schistosomiasis in Ikingwamanoti village, Shinyanga District, Tanzania.

**Methods:**

Urine samples were collected at baseline and follow-up time points from 282 school-aged children and examined microscopically for the presence of *S. haematobium* eggs. Malacological surveys involved collection of *Bulinus nasutus* every month from 30 sites. Snails were examined for patent infections. Global positioning system was used to map household distances from *S. haematobium* transmission sites, while water contact behavior was assessed using a questionnaire.

**Results:**

*Schistosoma haematobium* infection was observed to be prevalent among older children (12–14 years) compared to younger groups prior to treatment, but no significant difference in infection prevalence was observed at one-year. Boys were highly infected than girls at both time points. No spatial influence was observed between children’s infection and the distance from child’s residence to the nearby snail habitats nor was any significant association observed between children’s reported water contact behavior with *S. haematobium* infection. However, malacological surveys with cercarial shedding combined with GPS data detected significant variation among different water sources in the transmission of S*. haematobium* with children living in households near to ponds with high *B. nasutus* populations having the highest prevalence of infection.

**Conclusions:**

Interaction between malacological surveys with cercarial shedding combined with GPS mapping in endemic settings can help detection of transmission sources even in areas with complex transmission networks. Subsequent studies are needed to determine whether the combination of GPS mapping and parasitology screens can aid the detection of transmission hotspots across varied transmission settings to enhance schistosomiasis control programmes.

**Electronic supplementary material:**

The online version of this article (10.1186/s13071-018-3064-5) contains supplementary material, which is available to authorized users.

## Background

Schistosomiasis is a debilitating disease of humans caused by digenetic trematodes of the genus *Schistosoma* [1, 2]. Globally schistosomiasis affects more than 230 million people [3]. The disease has an extensive geographical distribution and is highly infective to people living in areas with limited access to safe water, sufficient sanitation and hygiene [4, 5] and adequate levels of appropriate health education [6]. Implementation of different schistosomiasis targeted controls strategies should mainly be determined from the local geographical diversities that enhance the nature of schistosomiasis transmission [7–10]. Human infection tends to vary with host immunity, water contact patterns, and geographical location, due to the presence and distribution of suitable snail intermediate hosts in water sources.

In most schistosomiasis-endemic areas design of targeted control measures and facilitating progress towards schistosomiasis elimination by adjustment of the ongoing schistosomiasis control interventions is necessary. By considering the focal geographical distribution and transmission of the disease, evaluation of the current schistosomiasis control interventions can occur.

In Tanzania, urogenital schistosomiasis is recognized to be endemic in Sukumaland in the south and southeast part of the Lake Victoria [11]. The disease is transmitted by *Schistosoma haematobium*, a digenetic trematode that develops into the human-infective stage within *Bulinus* snails that act as the intermediate hosts [12]. The infection is highly prevalent among school-aged children as reported in previous studies [1, 13], but infection is highly focally distributed, and the risk of acquiring infection by humans is determined by the presence of the compatible snail intermediate hosts that are responsible for parasite transmission [12, 14–16]. Studies on micro-geographical schistosomiasis transmission patterns reveal that there is discrepancy in infection intensities among households that is mainly associated with diverse water contact behaviors which interact with the limitation of transmission to particular sites due to the distribution of snail hosts [8, 17, 18]. In Shinyanga the transmission of, urogenital schistosomiasis is complex, occurring within networks of temporary ponds and streams. The water sources where snail intermediate hosts reside are mainly temporary pools and ponds that are created from dry river beds after the rainy season. They are the major water sources for domestic and animal use by the local population.

Here we examine micro-geographical patterns in transmission of, urogenital schistosomiasis, in this area with a complex network of potential transmission water bodies (ponds), by combining parasitological surveys of school-aged children with malacology surveys, behavioral questionnaires and GPS mapping of household, with the intention of determining their usefulness as tools for identifying transmission hotspots.

## Methods

### Study area and population

This study was carried out from October 2015 to July 2017 as a longitudinal study of school-aged children and snail populations in Ikingwamanoti village, Shinyanga Region. No prior preventative chemotherapy mass drug administration had been implemented in the area. Ikingwamanoti village is located at 03.92064°S, 33.12066°E and at an altitude of the area is approximately 1000 m above sea level. The region has two rainy seasons, the long rainy season from March to May/June and the short rainy season from November to December each year. *Wasukuma* is the pre-dominant ethnic group in the area.

Shinyanga is an area with black cotton soil with non-permanent streams that occasionally flow after rain. The local population gets water mainly from pools and ponds along dry river beds or man-made pools which are shared by domestic livestock. Boreholes are not common. Irrigation, especially along water sources is practiced, particularly during the dry season.

Economically, most of the inhabitants earn their living from small-scale agricultural and livestock farming (mainly cattle, goats and sheep). Livestock farming goes together with crop production (mainly maize, sorghum and rice), but relative contribution of livestock and crop production varies between individual households. Therefore, land and labor constitute the main components of the economic system, the basis of which is founded on family labor.

A total of 250 children (6–14 years-old) attending Ikingwamanoti Primary School were enrolled in the study. Children were followed-up three-weeks and one-year after treatment for schistosomiasis. A total of 30 water contact points in the village were surveyed for presence of *Bulinus* snails on a monthly basis.

### Parasitological investigation and treatment

Three consecutive urine samples were collected from all participating children pre-treatment, which took place during the dry season, and again three weeks later to assess treatment efficacy, and one-year later to assess re-infection. Ten millilitres of each urine sample were agitated and filtered through a 10 μm Nucleopore polycarbonate filter membrane (Whatman® Nuclepore™ UK) for counting of *S. haematobium* eggs. Immediately after the pre-treatment survey and after the one-year follow-up survey, all children were treated with a single dose of praziquantel (~40 mg/kg), using the standardized praziquantel dose pole [19].

### Water contact questionnaire surveys

A total of 250 school children attending Ikingwamanoti Primary School who were recruited into the study were interviewed by trained research assistants using a structured questionnaire in *Kiswahili* to record demographic information, water contact activities, water contact sites, frequency of water contact, time of day when water contact occurred and duration of water contact. The questionnaire was administered during the parasitological follow-up survey. Those who missed the questionnaire surveys were removed during data analysis.

### Malacological surveys

All water contact points in Ikingwamanoti village were identified in 2015 through observations and unstructured interviews with members of the community. Sites were selected on the basis of availability of water and observation of human or animal water contact activities. A total of 30 snail sampling sites for schistosomiasis transmission hot spots were determined and followed monthly from March 2016 to July 2017 to assess for the dynamics of snail intermediate hosts and their ability to transmit *S. haematobium*. Sites were grouped into snail habitats, each composed of one large pool or series of small pools. A hand-held Garmin GPSMAP 64sGPS was used to record the geographical coordinates for each habitat.

During the surveys, snails were collected by a single person using a hand-held scoop made of 2 mm wire mesh for a period of 10 min per site to quantify snail abundance. All snails collected were classified based on shell morphology to genus level, with provisional identification to species level. Snails were individually placed in wells filled with distilled water and then exposed under natural light for 2 h to initiate cercarial shedding. Using a dissecting microscope each snail was examined to determine presence of schistosome cercariae potentially infective to humans. *Schistosoma bovis* was transmitted in the area and cannot be differentiated from *S. haematobium* at the morphological level.

### Household mapping

**Fig. 1:**
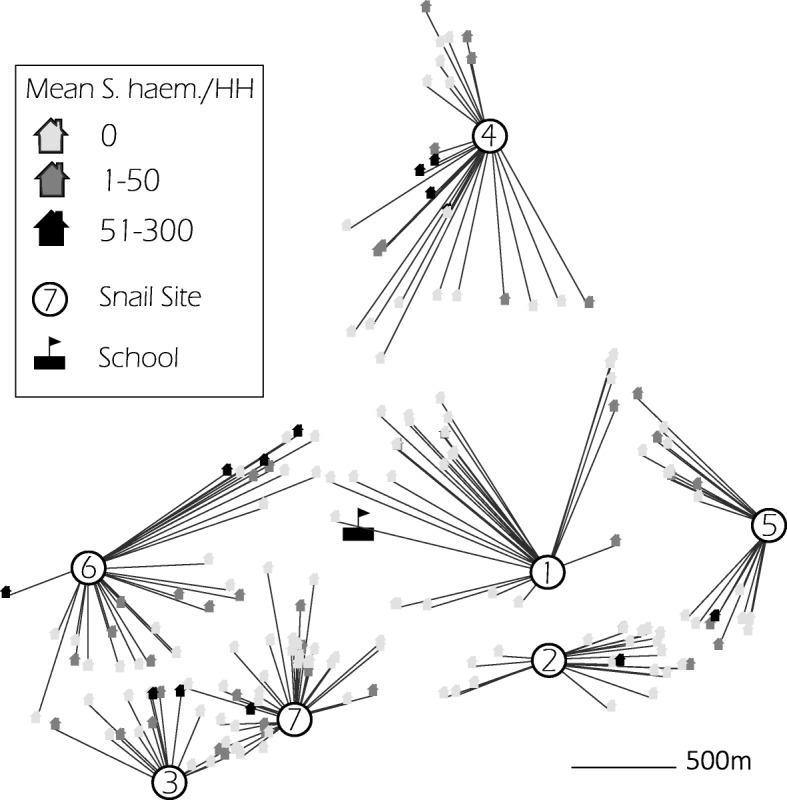
Household allocation to snail site and mean household intensity of infection of *S. haematobium* infection. The assignment of households to central coordinates of the nearest habitat is shown, as are the mean pre-treatment infection intensities, grouped according to no infection, light infection (1–49 eggs/10 ml of urine) or high infection intensity (≥ 50 eggs/10 ml of urine), recorded for each household

The households of each child attending Ikingwamanoti Primary School were mapped using a hand-held Garmin GPSMAP64s GPS. Household coordinates, along with snail sampling site coordinates, were imported in ArcView software. For each habitat, a central coordinate was assigned from those of the associated snail sampling sites, using visualization from satellite images to guide this assignment. The distances of school-children’s’ residence from these snail habitats were calculated and the children assigned to the nearest habitat (Fig. 1).

### Data analysis

The study data was analyzed using R v.3.2.1. Study participants were classified into three groups based on age (6–8 years; 9–11 years; and 12–14 years). The binomial regression model was used to calculate the odds ratios (OR) of infection and confidence intervals for age and sex during baseline and in re-infection prevalence. The re-infection model was controlled for infections not cleared by treatment by inclusion of the children’s infection status three weeks post-treatment (5.6%). To determine demographic and geographical predictors of *S. haematobium* infection, a logistic regression model was generated by including all parameters in a multivariate analysis controlled *a priori* for age and sex. In a stepwise manner, non-significant variables were removed to improve the model on the bases of Akaike information criterion (AIC) value resulting in the final model. Spearman’s rank correlation was used to assess the association between the prevalence of infection within children and the number of snails shedding potential human-infective cercariae. Each water contact activity was analysed independently in logistic regression models adjusted *a priori* for sex and age group. A *P*-value of 0.05 was considered statistically significant.

## Results

### Prevalence of *S. haematobium* infections by demographic characteristics

The prevalence of *S. haematobium* infection before treatment and one-year post-treatment was 34.8 and 16.8%, respectively. This represents a 51.7% reduction in the prevalence of *S. haematobium* infection after one round of single dose praziquantel treatment. Children aged 12–14 years had a significantly higher prevalence of *S. haematobium* infection at pre-treatment compared to younger age groups, but atone year post-praziquantel treatment, there was no significant differences in the prevalence of *S. haematobium* infection among age groups (Table 1). At the assessment of treatment efficacy, three weeks post-treatment, a considerable reduction of *S. haematobium* prevalence from 34.8 to 5.6% was observed.

**Table 1:** Demographic characteristics of school-aged children in relation to pre-treatment infection and one-year post-treatment re-infection prevalence of *S. haematobium*

Variable	*n*	Pre-treatment			Re-infection^a^		
		No. infected (%)	OR (95% CI)	*P*-value	No. re-infected (%)	OR (95% CI)	*P*-value
Sex							
Girls	146	37 (25.34)	1		7 (4.79)	1	
Boys	104	50 (48.08)	2.57 (1.49–4.50)	0.0001	35 (33.65)	8.95 (3.94–23.15)	0.0001
Age (years)							
6–8	87	17 (19.54)	1		12 (13.79)	1	
9–11	84	31 (36.90)	2.38 (1.19–4.91)	0.01	15 (17.85)	1.24 (0.49–3.11)	0.648
12–14	79	39 (49.37)	3.75 (1.88–7.73)	0.0001	15 (18.98)	1.17 (0.47–2.93)	0.734

^a^Model of re-infection prevalence was controlled for presence or absence of detectable infection 3-weeks post-praziquantel treatment

*Abbreviations*: *n* number enrolled, *CI* confidence interval

The sex-specific infection prevalence of *S. haematobium* at pre-treatment and at one-year re-infection is shown in Table 1. Pre-treatment, boys had a higher prevalence of *S. haematobium* infection (48.08%) compared to girls (25.34%). One-year post-treatment, the prevalence of *S. haematobium* infection in boys decreased to 33.65% but remained significantly higher compared to girls (4.79%) (Table1).

### Malacological surveys

A total of 4899 snails were collected from seven habitats sampled within the village. Overall, 132 snails (2.7%) shed schistosome cercariae. Although, the highest snail density was observed in Habitat 3 (Mwakasela), where 1678 snails were collected representing 34.25% of the total *Bulinus nasutus* collected in the village, this habitat had neither the highest number nor the highest prevalence of snails with patent infection, with only 22 (1.31%) shedding schistosome cercariae. The highest number and prevalence of snails with patent infection was observed in Habitat 5 (Jumanne), where 691 snails were collected, of which 48 (6.95%) shed schistosome cercariae. Habitat 4 (Mwamunonge) had the second highest number of snails collected (1450) and the second highest number of snails (45) with patent infections (3.1%). The habitat with the lowest transmission potential was Habitat 1 (Mwachumi) from which only one snail without patent infection was collected throughout the whole survey period (Table 2).

**Table 2:** Relative snail population numbers and patent infection with mammalian schistosomes by habitat

Habitat no.	Habitat name	No. of snails collected (%)	No. of snails infected (%)
1	Mwachumi	1 (0.02)	0 (0)
2	Miyu	49 (1.00)	1 (2.04)
3	Mwakasela	1678 (34.25)	22 (1.31)
4	Mwamunonge	1450 (29.60)	45 (3.1)
5	Jumanne	691 (14.10)	48 (6.95)
6	Mwamalago	149 (3.04)	1 (0.67)
7	Mwakangota	881 (17.98)	15 (1.70)
	Total	4899 (100)	132 (2.69)

### *Schistosoma haematobium* pre-treatment infection and snail habitats

**Fig 2:**
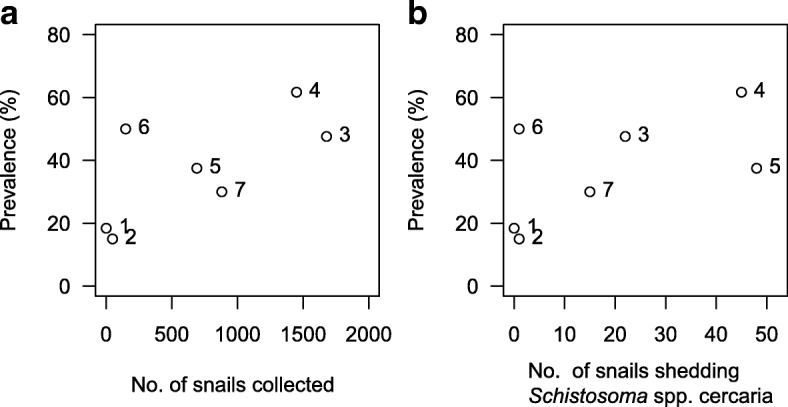
Prevalence of *S. haematobium* in school children in relation to intermediate host site, numbers and patent infections. Scatter plots of the prevalence of *S. haematobium* infection within school children assigned to their nearest snail habitat, indicated numerically, against the accumulated snail count for that habitat (**a**), or the accumulated number of snails that shed *Schistosoma* spp. cercariae (**b**)

Geographically, infection intensity (Fig. 1) and prevalence (Fig. 2) amongst school-children tended to be greatest near habitats with high numbers of snails shedding schistosome cercariae, indicating that the population size of the compatible snail intermediate hosts influences transmission levels to children living near a particular habitat. The prevalence of *S. haematobium* pre-treatment within school children was relatively highly correlated with the number of snails collected in the particular habitat (Fig. 2a) but not significantly so due to the low number of habitats (Spearman’s rho = 0.643, *P* = 0.139). A moderate correlation was also observed between children’s infection prevalence and the number of *Bulinus* snails shedding *Schistosoma* cercariae in the nearest snail habitats (Fig. 2b), but again this was not significant (Spearman’s rho = 0.523, *P* = 0.229).

To investigate whether the observed differences in snail numbers and *S. haematobium* infection rates between habitats were associated with differing infection prevalence among school children, a logistic regression model was built (controlling for age and sex) that included the nearest habitat to the child’s household. Habitat 1, for which only one uninfected snail was collected, and was the most centrally situated habitat (Fig. 1), was used as the reference. Although Habitat 3 had the highest observed number of snails, and high patent infection in snails, residence close to Habitat 3 was not significantly associated with a higher pre-treatment prevalence of *S. haematobium* infection in school children (OR: 3.16, 95% CI: 0.84–12.63, *P* = 0.093), compared to those whose households were closest to Habitat 1. However, residence close to Habitat 4, the habitat with the second highest snail population numbers, and patent infection, was significantly associated with higher pre-treatment *S. haematobium* infection prevalence (OR: 6.46, 95% CI: 2.14–21.88, *P* < 0.001) followed by residence close to snail Habitat 6, which was of borderline significance (*P* = 0.055) (Table 3). Classification as resident closest to Habitat 6 is the greatest anomaly amongst the trend between snail habitat and prevalence of infection in school children (Fig. 2).

**Table 3:** Pre-treatment logistic regression model of predictors of *S. haematobium* infection among school-aged children in Ikingwamanoti village

Variable^a^	OR (95% CI)	*P*-value
Sex		
Boys	2.64 (1.49–4.88)	0.001
Age (years)		
9–11	2.99 (1.39–6.68)	0.006
12–14	4.29 (2.00–9.55)	0.0001
Snail habitat		
Habitat 2	0.38 (0.08–1.57)	0.188
Habitat 3	3.16 (0.84–12.63)	0.093
Habitat 4	6.46 (2.14–21.88)	0.001
Habitat 5	2.89 (0.88–10.29)	0.087
Habitat 6	3.07 (1.01–10.29)	0.055
Habitat 7	1.34 (0.44–4.41)	0.609

^a^Female sex, aged 6–8 years and Habitat 1 were used as reference groups

Residence close to snail Habitat 2, Habitat 5 and Habitat 7 was not significantly associated with a higher infection prevalence of *S. haematobium* than residence closest to Habitat 1 (Table 3). There was no detectable spatial influence on prevalence of re-infection among school children living in Ikingwamanoti village.

### Water contact activities

From a water contact questionnaire, the most reported water contact activity conducted by children was fetching water, which was reported by 84% of the children; however, this involved collecting water from different water sources for their domestic use. No permanent water sources were available in the community, thus people depended on temporary water sources that were shared with livestock. Children responses to livestock watering were high (81.2%). Since the majority of the community members are pastoralists and tend to move from one place to another searching for water, 71.6% children reported having to crossed water in their daily activities. The frequency of paddy farming and other ways of water contact were 51.6% and 40%, respectively. The least reported water contact activities were swimming and fishing with frequencies of 24.4% and 14%, respectively.

A logistic regression model was built to assess various water contact activities performed by school children to confirm the risk of infection and re-infection with *S. haematobium* among school children. The model assessed different individual activities conducted by school children, controlling *a priori* for age and sex. No specific activity was observed to be a significant risk activity for S*. haematobium* infection (Table 4), and being aged 12–14 years (OR: 3.29, 95% CI: 1.57–7.16, *P* < 0.001) and being a boy (OR: 3.54, 95% CI: 1.57–7.74, *P* = 0.002) remained significant predictors of *S. haematobium* infection.

**Table 4:** Assessment of individual water contact activities in relation to *S. haematobium* prevalence amongst school-aged children in Ikingwamanoti village

Water contact activity	No. of individuals (%)	OR (95% CI)	*P*-value
Livestock watering	203 (81.2)	1.22 (0.60–2.58)	0.596
Fetching water	209 (83.6)	1.16 (0.52–2.69)	0.725
Swimming	60 (24)	0.85 (0.43–1.63)	0.627
Irrigation	95 (38)	0.99 (0.56–1.76)	0.979
Crossing water	179 (71.6)	1.13 (0.60–2.14)	0.715
Paddy farming	129 (51.6)	1.28 (0.72–2.26)	0.397
Other	99 (39.6)	1.33 (0.75–2.35)	0.325

Logistic regression models were adjusted *a priori* for age-group and sex. For each activity, the children who reported not participating in it were used as the reference

Logistic regression models were also used to assess where, when, frequency and time spent in water contact activities as risk factors for the transmission of *S. haematobium* among school-aged children. All models were adjusted *a priori* for age and sex and reduced in a stepwise manner removing insignificant variables. No individual activity was found to be a significant risk factor for S*. haematobium* infection (*P* > 0.05). Domestic activities, such as laundry and dish washing, along with bathing were mostly reported to be conducted at home and were not analysed (Additional file 1: Table S1). Although majority of the water contact activities were observed to be performed by the school children at different frequencies and time, no specific reported activity was demonstrated to be a significant risk factor for transmission of *S. haematobium* in school children in the village (Additional file 1: Table S1).

## Discussion

Schistosomiasis control approaches that are well adapted to epidemiological settings need to address the foremost risks associated with local transmission, in addition to applying the essential recommended schistosomiasis control with praziquantel mass drug administration as a foundation [20, 21]. Therefore, understanding the predictors for local *S. haematobium* transmission is critical. This study presents findings on the risk factors that are significantly associated with infection and re-infection among school aged children over duration of 12 months. Our findings indicated that sex, age, and proximity of residence to habitats with high population numbers of the intermediate snail host were independent determinant factors for *S. haematobium* infection among school-aged children. Reported water contact activities were not significantly associated with infection with *S. haematobium* in this study.

Male sex was a significant predictor of pre-treatment *S. haematobium* infection and re-infection in our study. It is expected that sex role differences had impact on the level of exposure to *S. haematobium* infection among boys than girls. It is possible that boys were more exposed to *S. haematobium* infection compared to girls due to their routine livestock caring, in which much time is spent searching for different pastures and water sources for watering, and since boys spent most of their time in the field, they utilize that time to swim after cattle watering (Additional file 2: Table S2). However, we found that implementation of a simple questionnaire was insufficient to significantly attribute this to infection prevalence. A higher prevalence of infection amongst boys is consistent with previous studies carried out elsewhere [22–24].

Pre-treatment, children aged 12–14 years had higher risk of *S. haematobium* infection than the youngest age group. This could be due to increased duration of water contact activities that expose them to a higher risk of contracting schistosomiasis, or their longer lifetime to accumulating the parasite without the development of resistance to infection. After 12 months of praziquantel treatment there were no significant differences in infection levels between age groups, as has been reported by previous studies [25–29]. This indicates that protective immunity has not developed and that changes in behavior are also not as significant a factor as time for accumulation of infection. Regular treatment is therefore necessary to maintain low levels of schistosomiasis infection and prevent development of disease morbidity within this community.

In this study, the children’s reported involvement in any particular water contact activity was not directly associated with *S. haematobium* infection. In the assessment of water contact activities among school-aged children as predictors of *S. haematobium* infection, our study demonstrated that only sex and age were significant risk factors for infection and not the type of water contact performed by children. This reflects that a simple knowledge, attitude and practice questionnaire approach is insufficient to provide the necessary information required to identify status of water contact performed by children in relation to schistosomiasis transmission. Engagement of in-depth water contact discussions is necessary to clearly determine time, where and when water contact activities are performed by children. Previous studies also observed that only age and sex (i.e. universal variables) were predictors of *S. haematobium i*nfection and not water contact activities [27].

Snail habitats containing infected snails has an impact on the magnitude of exposure as a risk factor for transmission of schistosomiasis [18]. The distance between households and snail habitats in the village were determined by the use of GPS. The use of GPS proved to be reliable for determination of specific locations as the source of transmission in this area, as confirmed by malacology surveys. The closest snail habitat to child’s household was a significant predictor of pre-treatment infection. However, there was no spatial influence on re-infection among the school children. Although previous studies have indicated that distance from homestead to an open water source is a determinant risk for *S. haematobium* infection [26], in our study it was the actual habitat that was closest that predicted infection prior to treatment, rather than the measured distance. School children mostly at risk of *S. haematobium* infection in the village were those whose households were located closer to snail habitats harboring the most abundant snail intermediate host populations, though not the highest prevalence of patently infected snail hosts. This may reflect that we were unable to identify *S. haematobium* from *S. bovis* cercariae at the morphological level; future studies need to employ molecular techniques to specifically differentiate *S. bovis* from *S. haematobium* cercariae, and to critically elucidate the transmission pattern of schistosome species existing within particular geographical settings. Other anomalies included assignment to Habitat 1, where only one uninfected snail was collected throughout the duration of the study, but infection prevalence amongst the children was almost 20%, and assignment to Habitat 6, for which 50% of the children were infected but only 149 snails, one patently infected, were collected. This may reflect mis-assignment of children to habitats, as distance to habitat may not capture fully actual sites of water contact. As our observations have confirmed that use of questionnaire alone in identifying predictors of *S. haematobium* transmission can be insufficient to reveal the reality of where transmission takes place, or the behaviors with highest risk for infection, linkage of GPS with in-depth water contact discussions may correct for the types of anomaly observed. However, the GPS tool was strong compared to the use of questionnaires, though it should be noted that not all recruited participants participated in the questionnaire exercise.

Although, the concurrent use of pre-treatment parasitological surveys with malacological surveys, and GPS enabled the detection of specific micro-geographical sites responsible for *S. haematobium* transmission in this area with a complex network of water sources, this was not apparent in the analysis of the one-year re-infection data. At one-year the majority of children were not infected. Therefore, under the current force of infection of, urogenital schistosomiasis occurring in the village observation of the spatial influence on re-infection was not possible, and it depended on collective exposure to *S. haematobium* infection over several years. Therefore, in endemic areas where transmission is low, or that have received several rounds of chemotherapy with praziquantel, parasitological and GPS surveys alone may be insufficient to detect on-going transmission sites, and detailed malacological surveys may be required.

## Conclusions

Our study shows that malacological surveys with cercarial shedding combined with GPS data detected variation among different water sources in the transmission of S*. haematobium* with children living in households near to ponds with high *B. nasutus* populations having the highest prevalence of infection. This approach can help determine specific transmission sources that exist within a complex transmission network for enhancement of targeted control strategies. The current commended schistosomiasis control initiatives adapted in many endemic countries as per WHO guidelines [21], rarely break the transmission of schistosome parasites within the endemic human communities, new and integrated interventions are required and predictors of transmission in local settings are required to best target these integrated interventions [30–33]. Subsequent studies are needed, combining the use of GPS data in identification of specific transmission sources across areas with differing transmission patterns, in order to validate the breadth of applicability of this approach. Additionally, upon identification, in-depth malacological studies may be required to determine further key aspects of transmission such as seasonality, if targeted malacological control interventions, in combination with the current preventative chemotherapy interventions, are to help the move towards schistosomiasis elimination.

## Additional files

Additional file 1: **Table S1.** Assessment within activity to determine where, when, frequency and duration of water contact activities in relation to *S. haematobium* infection in school children in Ikingwamanoti village. (DOCX 18 kb)

Additional file 2: **Table S2.** Breakdown of reported water contact activities by sex and age. (DOCX 14 kb)

## Abbreviations

AIC: Akaike information criterion
CI: confidence interval
GPS: Global Positioning System
HBREC: University of Cambridge Human Biology Research Ethics Committee
MRCC: Medical Research Coordination Committee
NIMR: National Institute for Medical Research
OR: odds ratio
Rho: Spearman’s rank correlation coefficient
WHO: World Health Organization
