# Supplementary material for: Geographical and behavioral risks associated with Schistosoma haematobium infection in an area of complex transmission
Source: Parasit Vectors. 2018 Aug 25;11:481. doi: 10.1186/s13071-018-3064-5 (PMC6109322; doi:10.1186/s13071-018-3064-5)
Supplement: Supplementary file 1 — Table S1. Assessment within activity to determine where, when, frequency and duration of water contact activities in relation to S. haematobium infection in school children in Ikingwamanoti village. (DOCX 18 kb) [file 13071_2018_3064_MOESM1_ESM.docx]

**Table S1: Assessment within activity to determine WHERE, WHEN, FREQUENCY and duration of water contact activities in relation *to S. haematobium* infection in school children in Ikingwamanoti village.**

| **ACTIVITY** | **Categories** | **N (%)** | **OR(95% CI)** | **P-VALUE** | |
| --- | --- | --- | --- | --- | --- |
| Livestock | **Having livestock** | 143 (57.2) | 0.22 ( 0.60- 2.58) | 0.596 |  |
|  | **Where livestock watering occurs** |  |  |  |  |
|  | Home |  | 3.19 (0.61 -18.48) | 0.168 |  |
|  | Modern wells |  | 1.22 ( 0.22 -5.40) | 0.802 |  |
|  | Traditional wells |  | 0.66 (0.29- 1.50) | 0.316 |  |
|  | Pond |  | 1.71 (0.82 - 3.65) | 0.161 |  |
|  |  |  |  |  |  |
| Dish washing | **Dishwashing**  **Where dish washing is done** | 159 (63.6) |  |  |  |
|  | Home |  | 0.77 (0.29 -2.02) | 0.587 |  |
|  | Pond |  | 1.60 ( 0.05 - 46.62) | 0.756 |  |
| Water collection | **Collecting water** | 209 (83.6) | 1.15 (0.52 -2.67) | 0.734 |  |
|  | **Where water is collected** |  |  |  |  |
|  | Traditional well |  | 0.20 (0.66 -2.21) | 0.551 |  |
|  | Pond |  | 0.99 (0.41 -2.33) | 0.980 |  |
| Swimming | **Swimming** | 60 (24) | 3.69 (1.85 -7.62) | 0.627 |  |
| Laundry | **Laundry**  **Where laundry is done** | 46 (18.4) |  |  |  |
|  | Pond |  | 0.97 (0.47 – 1.97) | 0.936 |  |
|  | **Frequency of laundry** |  |  |  |  |
|  | Daily |  | 1.17 (0.63 - 2.20) | 0.650 |  |
|  | **Time of a day for laundry** |  |  |  |  |
|  | After lunch to sunset |  | 0.96 (0.50- 1.89) | 0.918 |  |
| Fishing | **Fishing**  **Time spent in fishing** | 35 (14) |  |  |  |
|  | Morning to lunch |  | 1.65 ( 0.69- 4.02) | 0.263 |  |
|  | After lunch to sunset |  | 0.72 (0.09 -4.19) | 0.720 |  |
|  |  |  |  |  |  |
| Irrigation | **Doing irrigation** | 95 (38) | 3.74( 0.27 -106.73) | 0.346 |  |
|  | **Frequency of irrigation** |  |  |  |  |
|  | 1-2 times per week |  | 1.59 (0.15 – 18.09) | 0.694 |  |
|  | Daily |  | 1.44 (0.19- 13.92) | 0.728 |  |
|  | **Time spent for irrigation** |  |  |  |  |
|  | Morning to lunch |  | 0.64(0.07 - 4.88) | 0.671 |  |
|  | After lunch to sunset |  | 0.49(0.05 - 4.13) | 0.524 |  |
| Bathing | **Place of bath** |  |  |  |  |
|  | Pond |  | 1.10 (0.31 3.94) | 0.876 |  |
|  |  |  |  |  |  |
|  | **Frequency of bathing** |  |  |  |  |
|  | Twice per day |  | 1.12 (0.61 -2.09) | 0.718 |  |
| Crossing water | **Crossing water** | 179 (71.6) | 0.46 (0.05- 4.24) | 0.466 |  |
|  | **Place of water contact** |  |  |  |  |
|  | Home |  | 3.37 (0.33- 35.89) | 0.284 |  |
|  | Traditional wells |  | 2.07(0.21- 21..07) | 0.516 |  |
|  | Paddy farms |  | 2.84 (0.28- 29.56) | 0.354 |  |
| Other ways of contacting water | **Time of a day for water contact** |  |  |  |  |
|  | Evening |  | 1.30( 0.40 3.93) | 0.645 |  |
|  | Morning |  | 1.35 (0.70- 2.60) | 0.368 |  |
|  | Afternoon |  | 1.03( 0.34- 2.94) | 0.958 |  |
| Paddy farming | **Working in paddy farms** | 129 (51.6) | 1.28(0.72 -2.26) | 0.397 |  |
