# Supplementary material for: Geographical and behavioral risks associated with Schistosoma haematobium infection in an area of complex transmission
Source: Parasit Vectors. 2018 Aug 25;11:481. doi: 10.1186/s13071-018-3064-5 (PMC6109322; doi:10.1186/s13071-018-3064-5)
Supplement: Supplementary file 2 — Table S2. Breakdown of reported water contact activities by sex and age. (DOCX 14 kb) [file 13071_2018_3064_MOESM2_ESM.docx]

**Table S2 Breakdown of reported water contact activities by sex and age**

|  | **Male(n = 104)** | | | **Female (n = 146)** | | |
| --- | --- | --- | --- | --- | --- | --- |
| **Water contact activity** | **6-8yrs**  **n (%)** | **9-11yrs**  **n (%)** | **12-14yrs**  **n (%)** | **6-8yrs**  **n (%)** | **9-11yrs**  **n (%)** | **12-14**  **n (%)** |
| Livestock watering  Domestic activities* | 23 (22.1)  34 (32.7 | 27 (25.9)  29 (27.8) | 34 (32.7)  40 (38.5) | 43 (29.5)  101(69.2) | 44 (30.1)  93 (63.7) | 32 (21.9)  65 (44.5) |
| Fetching water | 26 (25) | 27 (25.9) | 35 (33.7) | 39 (26.7) | 44 (30.1) | 38 (26) |
| Swimming | 5 (4.8) | 9 (8.6) | 10 (9.6) | 16 (10.9) | 15 (10.3) | 5 (3.4) |
| Irrigation | 10 (9.6) | 10 (9.6) | 15 (14.4) | 18 (12.3) | 25 (17.1) | 17 (11.6) |
| Crossing water | 24 (23.1) | 21 (20.2) | 34 (32.7) | 32 (21.9) | 38 (26) | 30 (20.5) |
| Paddy farming | 9 (8.6) | 17 (16.3) | 24 (23.1) | 24 (16.4) | 30 (20.5) | 25 (17.1) |
| Other | 10 (9.6) | 12 (11.5) | 18 (17.3) | 23 (15.7) | 19 (13) | 17 (11.6) |

***** Includes both laundry and dish washing.
